# Subjective memory complaints in Italian elderly with mild cognitive impairment: implication of psychological status

**DOI:** 10.1007/s10072-016-2553-6

**Published:** 2016-03-30

**Authors:** Cinzia Giuli, Paolo Fabbietti, Cristina Paoloni, Mirko Pensieri, Fabrizia Lattanzio, Demetrio Postacchini

**Affiliations:** Unit of Geriatrics, Italian National Research Center on Aging, INRCA, IRCCS, Contrada Mossa, 63900 Fermo, Italy; Biostatistical Centre, Italian National Research Center on Aging (INRCA), Ancona, Italy; Scientific Direction, Italian National Research Center on Aging, INRCA, Ancona, Italy

**Keywords:** Subjective memory complaints, Elderly, Mild cognitive impairment, Cognitive training, Depression, Italy

## Abstract

Subjective cognitive and memory complaints (SMC) are common in later life and are considered an indicator for progression to cognitive decline. The aim of the present study was to identify the relationship among SMC, neuropsychiatric symptoms and psychological aspects in elderly subjects with mild cognitive impairment (MCI) as well as to analyse the effect on SMC of a comprehensive cognitive training. Data from a sample of 94 patients enrolled in ‘My Mind Project’ (Grant No. 154/GR-2009-1584108) were collected. The study evidenced that depression was a significant predictor of SMC and that after the training, the number of subjects with SMC was significantly reduced in the experimental group in comparison to the control one. These results suggest that the participation in cognitive stimulation protocols may improve the perception of SMC in subjects with MCI.

## Introduction

The study of the role played by subjective memory complaints (SMC) in later life is a key topic. In particular, SMC have been considered a main criterion for evaluation and classification of mild cognitive impairment (MCI) [1] and are recognized by some authors as indicator for progression to dementia [2]. Contrarily, as reported in a recent study, some authors, suggested that SMC are not an essential criterion for MCI [3]. Yates et al. appointed that the possible reason for this discrepancy could include individual variation of elderly people in adapting to cognitive change where some individuals may not perceive such changes as significant or requiring actions. In particular, these authors evidenced that progression to dementia is better predicted by SMC than by global cognitive impairment or by domain-specific cognitive decline [3].

Elderly people are usually affected by perception of loss of cognitive functions than they were young [4]. In particular, SMC are common in the healthy older population, but often they may not be experienced by elderly with cognitive decline [1]. Some authors have stated that SMC may influence the relationship between mood and cognitive functioning [3] and may have a negative effect on well-being as complaints might reflect health and psychological disorders such as depression, perceived stress, and anxiety [5, 6].

Memory knowledge and objective memory performance can be effectively improved in old age through specific trainings, which include the use of mnemonic strategies [7, 8].

Given this background, the aims of this study were:

1. To analyse the predictors of SMC at baseline, taking into account the relationship among SMC, neuropsychiatric symptoms and psychological status in elderly patients with MCI;
2. To evaluate the immediate impact of a comprehensive intervention on SMC (phase of follow-up 1).

## Materials and methods

### Subjects and recruitment

Preliminary analyses were conducted in 94 Italian community-dwelling elderly subjects with multidomain MCI living in the Marche region, enrolled in the study ‘My Mind Project: The effects of cognitive training for elderly’ from the Evaluation of Alzheimer’s Unit at the INRCA Hospital in Fermo. Eligibility was determined after a complete medical and neuropsychological examination to assess inclusion/exclusion criteria, as indicated in Casoli et al. [9]. The inclusion criteria included: age 65 years or older, availability during the training and testing phases, and presence of a caregiver. Exclusion criteria included: presence of serious medical or psychiatric conditions and sensori-motor deficits that would prevent participation in training, and presence of neurodegenerative disorders.

The study used a prospective randomized intervention protocol for the assessment of the effect of a comprehensive cognitive training. After the enrolment, subjects were randomly assigned to the experimental group (EG), whose members received the intervention (the detailed description is reported below) or to the control group (CG). Subjects with scores greater than or equal to 25 on the Memory Assessment Complaint Questionnaire (MAC-Q) of Crook were classified as ‘Complainers’ while the other subjects were defined as ‘Non-complainers’. Four groups were identified: ‘Complainers’ and ‘Non complainers’ included into EG and CG.

All subjects were tested at baseline and at the end of intervention (follow-up) using a neuropsychological test battery including assessments of overall cognitive status, memory and learning processes, attention, language and executive functions, functional status, psycho-social aspects, lifestyle characteristics [10].

### Instruments

#### Assessment of SMC

The MAC-Q was used to assess participants’ memory complaints. It is a questionnaire for the assessment of daily activities and overall memory functioning comparing present moment to past.

#### Assessment of mood status

Researchers used the Geriatric Depression Scale (GDS–30) of Yesavage to measure participants’ mood status.

#### Assessment of perceived stress

Participants’ perceived stress levels were measured using the Perceived Stress Scale (PSS) of Cohen. It comprises 14 items and asks about feelings and thoughts during the last month.

#### Assessment of functional status

The Activities of Daily Living (ADL) of Katz and Instrumental Activities of Daily Living (IADL) of Lawton and Brody were used to assess participants’ functional status.

### Intervention

The EG received an individual comprehensive multi-modal intervention that included restorative and compensatory cognitive training, which consists of learning strategies for orientation, memory, categorisation and clustering. Because MCI subjects often present symptoms of anxiety, stress and/or depression due to their consciousness of cognitive decline, the potential influence of SMC was evaluated as they may affect the perception of cognitive decline. Some practical and compensation strategies were taught as well as aids and psychological support for stress and mood disease management to improve performance and psycho-education about memory loss. Advice and education on a healthy lifestyle was also provided. All participants were asked to perform cognitive and metacognitive at-home exercises each day. The intervention consisted of 10 sessions of about 45 min, once a week.

### Statistical analysis

Data were expressed as mean ± standard deviation (continuous variables) or as percentage (categorical variables). Statistical comparisons were performed using the *t*-Student test or by Chi square test to compare Complainers and Non-complainers in EG and CG at baseline and follow-up. To obtain an estimate of the independent association between study variables and outcomes, variables significantly distinguishing groups in preliminary analysis were entered into a logistic regression model; Pearson’s coefficient was used to assess correlation among variables. The significance was accepted for *p* < 0.05. All analyses were performed using SPSS V19.0 Statistical Software Package for Windows.

## Results

Comparisons of socio-demographic data and neuropsychological profile between EG and CG ‘Complainers’ and ‘Non complainers’ at baseline and follow up were reported in Tables 1, 2.

**Table 1:** Characteristics of the experimental (EG) and the control (CG) group at baseline

	Baseline					
	EG			CG		
	Non complainers	Complainers	*p*	Non complainers	Complainers	*p*
	*N* = 8	*N* = 38		*N* = 18	*N* = 30	
Age (years)	75.88 ± 5.24	75.95 ± 6.53	0.974	76.67 ± 6.32	76.40 ± 5.58	0.267
Gender (female)	5 (62.5 %)	25 (65.8 %)	0.859	11 (61.1 %)	19 (63.3 %)	0.878
Marital status						
Married/cohabiting	7 (87.5 %)	22 (57.9 %)	0.115	14 (77.8 %)	22 (73.3 %)	0.728
Widowed	1 (12.5 %)	16 (42.1 %)		4 (22.2 %)	7 (23.3 %)	
Other	0 (0.0 %)	0 (0.0 %)		0 (0.0 %)	1 (3.3 %)	
Education (years)	5.75 ± 1.38	6.97 ± 4.22	0.426	5.88 ± 3.96	4.90 ± 2.21	0.273
GDS	6.75 ± 3.69	11.18 ± 6.41	<0.05	6.33 ± 3.71	10.86 ± 4.92	0.001
PSS	18.25 ± 7.90	20.00 ± 7.76	0.581	15.33 ± 6.29	20.76 ± 7.46	0.01
ADL	5.87 ± 0.35	5.81 ± 0.39	0.681	6.00 ± 0.00	5.73 ± 0.44	<0.05
IADL	8.00 ± 0.00	7.31 ± 0.93	<0.05	7.55 ± 0.70	7.46 ± 0.89	0.705

**Table 2:** Characteristics of the experimental (EG) and the control (CG) group at follow-up

	Follow-up					
	EG			CG		
	Non complainers	Complainers	*p*	Non complainers	Complainers	*p*
	*N* = 25	*N* = 21		*N* = 12	*N* = 36	
Age (years)	76.08 ± 7.41	76.29 ± 4.84	0.914	79.00 ± 4.72	75.89 ± 5.91	0.077
Gender (Female)	17 (68.0 %)	13 (61.9 %)	0.665	6 (50.0 %)	24 (66.7 %)	0.302
Marital status						
Married/cohabiting	17 (58.6 %)	12 (47.1 %)	0.447	10 (83.3 %)	26 (72.2 %)	0.686
Widowed	8 (41.4 %)	9 (52.9 %)		2 (16.7 %)	9 (25.0 %)	
Other	0 (0.0 %)	0 (0.0 %)		0 (0.0 %)	1 (2.8 %)	
Education (years)	6.16 ± 3.37	7.47 ± 4.42	0.271	5.83 ± 3.95	5.08 ± 2.64	0.549
GDS	8.52 ± 5.71	11.28 ± 6.13	0.124	7.41 ± 5.82	11.36 ± 4.54	<0.05
PSS	16.60 ± 8.42	20.57 ± 7.43	0.097	16.58 ± 9.29	21.08 ± 8.46	0.156
ADL	6.00 ± 0.00	5.61 ± 0.49	<0.001	6.00 ± 0.00	5.77 ± 0.42	0.222
IADL	7.44 ± 1.00	7.42 ± 0.74	0.965	7.58 ± 0.79	7.47 ± 0.84	0.111

At baseline, 72.3 % of MCI subjects had SMC. In particular, Complainers showed significant differences in comparison to Non-complainers in psychological and neuropsychiatric symptoms (mood and perceived stress), functional status (ADL, IADL) areas. In the whole sample at baseline, the GDS score was 6.46 (± 3.63) and the PSS score was 16.23 (± 6.80) for Non-complainers, whereas the scores for Complainers were significantly higher (*p* < 0.05) with a GDS score of 11.04 (± 5.76) and a PSS score of 20.33 (± 7.58).

A multiple logistic regression analysis using the baseline data showed that the predictor of SMC in elderly with MCI was the depression status. Using the Hosmer–Lemeshow goodness of fit test, the results showed *χ*^2^ = 10.342, *p* = 0.242, adjusting for gender, age, and score of the BADL, IADL, and PSS (Table 3).

**Table 3:** Logistic regression analysis at baseline

Dependent variables	OR	E.S.	*p*	95 % CI	
				Lower	Upper
Age	0.961	0.049	0.415	0.873	1.058
Gender	1.951	0.580	0.249	0.626	6.080
PSS	1.017	0.043	0.693	0.934	1.107
GDS	1.194	0.078	<0.05	1.025	1.391
ADL	0.337	1.122	0.332	0.037	3.038
IADL	0.664	0.416	0.325	0.294	1.502

After intervention, we observed a reduction of subjects with SMC in EG while no differences were found in CG (Tables 1, 2).

## Discussion

Our preliminary results evidenced that subjects with depression have higher risk to have SMC, which is recognized as indicator for eventual progression to dementia [2, 6]. The relationship between SMC and depression in MCI is controversial and not fully clarified [11], thus also in our study further analysis are necessary. Indeed, both mood and cognitive decline could have a mutual relationship with SMC [1]. Some authors evidenced that SMC are more related to depression than objective cognitive decline and recently they appointed that SMC might play an important role on the relationship between MCI and mood [3]. These authors concluded that SMC in MCI could be a function of neuropsychiatric symptoms (such as depression) rather than being related to objective cognitive functions. In this our preliminary study we found that depression is a significant predictor of SMC. We choose to finalize intervention for MCI subjects also to psychological support for stress and mood disease management, as well as training to improve cognitive performances by means of the use of memory strategies which can compensate perceived memory failures [8]. Therefore, a metacognitive and motivational approach which improve self-esteem for elderly with MCI is necessary during cognitive training, as also indicated by other authors [4]. This study evidenced that the enhancement of cognitive functions is not sufficient to improve confidence of the elderly about their performance, because often they have many difficulties in modifying motivational attitudes. For this reason, often the beneficial effect of intervention may be limited in duration.

An interesting result is that EG had positive effects in terms of decrease of SMC. Our findings showed that the multidimensional and comprehensive intervention, as described above, could be effective for elderly people, in particular in terms of improvement in self perception of memory loss and SMC. We also observed improvement on some memory and other cognitive performances in EG as indicated in Balietti et al. [10], but this relationship will be deeply studied. Other authors [7] found that this kind of intervention can enhance the overall cognitive functioning and encoding as well as recalling of new verbal information of older adults with SMC with a durable effect in memory [12].

In conclusion, taking into account that subjects with depression and SMC are at high risk of dementia, the identification of these groups is important to analyze factors and interventions that could possibly contribute to the prevention of cognitive decline and symptoms related to cognitive deficits (such as depressive mood), as also indicated by other authors [13].

Further investigation is required to define more reliable factors, including biomarkers, to discriminate the relationship between depression and SMC and to identify the long-term effects of intervention on psychological status and neuropsychiatric symptoms.
